# Antineutrophil Cytoplasmic Antibody-Negative Eosinophilic Granulomatosis With Polyangiitis in a Pediatric Patient

**DOI:** 10.7759/cureus.105160

**Published:** 2026-03-13

**Authors:** Joseph O Odeyemi, Apeksha Sathyaprasad

**Affiliations:** 1 Internal Medicine/Pediatrics, University of Kansas School of Medicine-Wichita, Wichita, USA; 2 Pediatric Pulmonology, University of Kansas School of Medicine-Wichita, Wichita, USA

**Keywords:** biologic treatment, churg-strauss syndrome, eosinophilic granulomatosis polyangiitis, leucocytoclastic vasculitis, severe asthma

## Abstract

Eosinophilic granulomatosis with polyangiitis (EGPA) is a rare pediatric vasculitis that can be difficult to diagnose, especially when antineutrophil cytoplasmic antibody (ANCA) is negative. We describe the case of a 10-year-old girl with severe asthma, recurrent leukocytoclastic vasculitis, peripheral eosinophilia, and pulmonary nodules. Infectious and immunologic workup was unremarkable, and skin biopsy confirmed eosinophilic vasculitis. She was diagnosed with ANCA-negative EGPA based on the 2022 American College of Rheumatology/European Alliance of Associations for Rheumatology criteria and started on high-dose corticosteroids with plans for mepolizumab maintenance. This case highlights the diagnostic challenges in pediatric EGPA, the masking effects of biologic therapy, and the importance of multidisciplinary evaluation for early recognition and optimal management.

## Introduction

Eosinophilic granulomatosis with polyangiitis (EGPA) is a rare systemic vasculitis affecting small- to medium-sized vessels, characterized by asthma, peripheral eosinophilia, and variable organ involvement, including pulmonary, cutaneous, cardiac, and neurologic manifestations [1]. Although predominantly described in adults, pediatric EGPA is increasingly recognized, with unique clinical features that may complicate timely diagnosis [2]. Children with EGPA often present with prominent asthma and eosinophilia, but systemic vasculitic manifestations can be subtle, leading to delayed recognition [1].

Antineutrophil cytoplasmic antibodies (ANCAs) are detectable in approximately 35-40% of adult EGPA cases, but ANCA-negative disease is more common in pediatric patients and may be associated with a higher frequency of cardiac and pulmonary involvement [1,3,4]. Therefore, the absence of ANCA can present a diagnostic challenge, as it may obscure early recognition and delay initiation of appropriate immunosuppressive therapy. Early diagnosis is critical because systemic involvement can progress rapidly and may result in significant morbidity if untreated [2].

We report the case of a 10-year-old female with moderate persistent asthma, recurrent leukocytoclastic vasculitis, and pulmonary imaging abnormalities, ultimately diagnosed with ANCA-negative EGPA. This case highlights the complexity of diagnosing EGPA in pediatric patients and underscores the importance of a multidisciplinary evaluation, including rheumatology, pulmonology, and allergy/immunology, to facilitate early recognition and management.

## Case presentation

A 10-year-old female had a history of moderate persistent asthma and allergic rhinitis for several years. Her asthma had been managed with high-dose inhaled corticosteroid/long-acting beta-agonist combination therapy and a leukotriene receptor antagonist, while her allergic rhinitis was treated with oral antihistamines and intranasal corticosteroids. Despite good adherence, her asthma was not well controlled, prompting initiation of omalizumab, which did not improve symptoms. She was subsequently switched to mepolizumab 40 mg monthly, resulting in significant improvement. However, due to social barriers, mepolizumab was discontinued for approximately six months, during which she experienced multiple asthma exacerbations requiring systemic corticosteroids and hospitalizations.

Additionally, the patient had a history of a recurrent purpuric rash on her lower extremities. A skin biopsy obtained approximately one year before the recent exacerbations demonstrated leukocytoclastic vasculitis with extravascular eosinophilia, supporting systemic eosinophilic inflammation. Initial laboratory evaluation, including ANCA, antinuclear antibodies, coagulation studies, and urinalysis, was largely unremarkable. However, she demonstrated elevated peripheral eosinophils off corticosteroids, mild elevations in inflammatory markers (erythrocyte sedimentation rate and C-reactive protein), with mildly elevated rheumatoid factor levels and markedly increased Immunoglobulin E levels (Table 1). Notably, her peripheral eosinophil counts were often normal during hospitalizations when she was receiving systemic corticosteroids for asthma exacerbations, highlighting the potential masking effect of prior therapy on disease expression. Comprehensive hematologic and immunologic evaluation, including peripheral blood smear evaluation, uric acid, lactate dehydrogenase, and flow cytometry, along with infectious testing for other causes of hypereosinophilia, was unremarkable. Serum tryptase was within normal limits, making systemic mastocytosis less likely.

**Table 1:** Key laboratory findings.

Laboratory test	Result/Finding	Reference range	Interpretation
Peripheral eosinophil count (on steroids)	0–0.1 × 10⁹/L	0–0.1 × 10⁹/L	Normal
Peripheral eosinophil count (off steroids)	2.34 × 10⁹/L	0–0.1 × 10⁹/L	High
Erythrocyte sedimentation rate	28 mm/hour	0–20 mm/hour	High
C-reactive protein	7.8 mg/L	<5 mg/L	High
Immunoglobulin E	24,094 IU/mL	12–708 IU/mL	High
Serum tryptase	2.6 ng/mL	0–11.5 ng/mL	Normal
Immunoglobulin A	48.1 mg/dL	50–400 mg/dL	Low
Immunoglobulin G	1,150 mg/dL	700–1,600 mg/dL	Normal
Complement C3	135 mg/dL	90–180 mg/dL	Normal
Complement C4	18.9 mg/dL	10–40 mg/dL	Normal
Rheumatoid factor	22.2 IU/mL	<14 IU/mL	High

**Figure 1:**
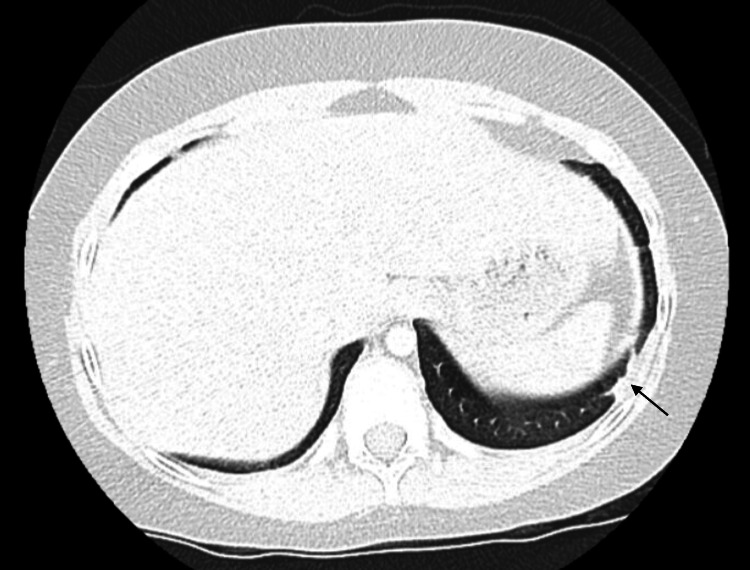
CT scan of the chest showing a subpleural nodule.

A CT scan of the chest demonstrated scattered subpleural nodules, ground-glass opacities, and partial right middle lobe collapse/atelectasis, without lymphadenopathy (Figure 1). Pulmonary function testing showed reversible obstructive lung disease with normal diffusing capacity of the lungs for carbon monoxide.

Based on these findings and the 2022 American College of Rheumatology/European Alliance of Associations for Rheumatology (ACR/EULAR) classification criteria, she was diagnosed with ANCA-negative EGPA. She was started on high-dose corticosteroids with plans to transition to high-dose mepolizumab 300 mg monthly for maintenance therapy.

## Discussion

Pediatric EGPA is uncommon and may be underrecognized due to symptom overlap with more prevalent pediatric conditions, including severe asthma, Henoch-Schönlein purpura, hypereosinophilic syndromes, drug-induced eosinophilia, and parasitic infections [1,2].

ANCA negativity is observed in around 75% of pediatric EGPA cases and is typically associated with eosinophilic tissue infiltration and prominent pulmonary and cardiac involvement, rather than the classic vasculitic manifestations seen in ANCA-positive disease [3,4]. In our patient, the recurrent purpuric rash and histologically confirmed leukocytoclastic vasculitis with extravascular eosinophilia were pivotal in distinguishing systemic EGPA from isolated allergic disease or other hypereosinophilic conditions. Her pulmonary findings, including subpleural nodules, ground-glass opacities, and reversible obstructive lung disease, further supported a multisystem process. These findings are consistent with prior reports indicating that pediatric ANCA-negative EGPA often presents with prominent pulmonary and tissue eosinophilia [1,3,4].

The patient’s prior asthma treatment with corticosteroids and biologics (omalizumab and mepolizumab) temporarily masked her symptoms and peripheral eosinophilia, complicating timely recognition. This phenomenon has been documented in adult and pediatric populations, emphasizing the need to review historical laboratory data and tissue biopsy findings when systemic eosinophilic disease is suspected [5,6]. High-dose corticosteroids remain the cornerstone of induction therapy, while biologics targeting interleukin-5, such as mepolizumab, can provide steroid-sparing maintenance therapy and help control eosinophilic inflammation.

This case highlights the value of a multidisciplinary approach, involving pulmonology, rheumatology, and immunology, to facilitate comprehensive evaluation and treatment. Differential diagnosis included other hypereosinophilic syndromes, systemic mastocytosis, and primary immunodeficiency, all of which were ruled out through laboratory testing. The application of the 2022 ACR/EULAR classification criteria enabled standardized diagnosis even in an ANCA-negative pediatric patient, demonstrating the utility of updated classification tools in clinical practice.

According to the ACR/EULAR classification criteria for EGPA, a score of ≥6 is required for classification, along with a diagnosis of small- or medium-vessel vasculitis and exclusion of potential mimics. It is important to note that these criteria are intended for classification rather than definitive diagnosis and may perform poorly in distinguishing EGPA from other hypereosinophilic syndromes. In our patient, the criteria were applied as follows: clinically, she had obstructive airway disease (+3), while nasal polyps and mononeuritis multiplex were absent; in laboratory and histologic evaluation, she had a blood eosinophil count ≥1 × 10⁹/L (+5) and extravascular eosinophil-predominant inflammation on biopsy (+2), with negative c-ANCA/PR3-ANCA and no hematuria. This resulted in a total score of 10, exceeding the threshold of 6 required for classification.

Compared with previous pediatric cases, this patient illustrates the diagnostic challenge of ANCA-negative EGPA, particularly in the context of biologic therapy and masked laboratory abnormalities. Early recognition and treatment are critical to prevent progression of systemic involvement and reduce morbidity. This report adds to the growing literature on pediatric ANCA-negative EGPA and emphasizes the importance of integrating clinical, histologic, and laboratory data in challenging cases.

## Conclusions

ANCA-negative pediatric EGPA can manifest with recurrent or difficult-to-control asthma, peripheral eosinophilia, and cutaneous vasculitis, posing a diagnostic challenge. This case highlights the importance of considering systemic eosinophilic disorders in children whose asthma is atypical or refractory to standard therapy, especially when accompanied by extrapulmonary manifestations. Comprehensive evaluation, including review of prior laboratory trends, tissue biopsy, and imaging, along with close collaboration between pulmonology, rheumatology, and immunology, is essential for accurate diagnosis and prompt initiation of therapy. Early recognition and management are critical to prevent progression of organ involvement, reduce morbidity, and optimize long-term outcomes. Clinicians should remain vigilant for subtle signs of systemic disease in pediatric patients with severe asthma and persistent eosinophilia, as early intervention can substantially alter the disease course and improve quality of life.
